# Adopting a healthy lifestyle when pregnant and obese – an interview study three years after childbirth

**DOI:** 10.1186/s12884-016-0969-x

**Published:** 2016-07-30

**Authors:** Anna Dencker, Åsa Premberg, Ellinor K. Olander, Christine McCourt, Karin Haby, Sofie Dencker, Anna Glantz, Marie Berg

**Affiliations:** 1 Centre for Person-Centred Care (GPCC), University of Gothenburg, Gothenburg, Sweden; 2 Institute of Health and Care Sciences, Sahlgrenska Academy, University of Gothenburg, Gothenburg, Sweden; 3 Centre for Maternal and Child Health Research, City University London, London, UK; 4 Antenatal Health Care, Primary Health Care, Research and Development Unit, Närhälsan, Gothenburg, Sweden; 5 Primary Health Care, Närhälsan, Gothenburg, Sweden

**Keywords:** Body mass index, Obesity, Gestational weight gain, Lifestyle intervention, Antenatal health care, Interview study

## Abstract

**Background:**

Obesity during pregnancy is increasing and is related to life-threatening and ill-health conditions in both mother and child. Initiating and maintaining a healthy lifestyle when pregnant with body mass index (BMI) ≥ 30 kg/m^2^ can improve health and decrease risks during pregnancy and of long-term illness for the mother and the child. To minimise gestational weight gain women with BMI ≥ 30 kg/m^2^ in early pregnancy were invited to a lifestyle intervention including advice and support on diet and physical activity in Gothenburg, Sweden.

The aim of this study was to explore the experiences of women with BMI ≥ 30 kg/m^2^ regarding minimising their gestational weight gain, and to assess how health professionals’ care approaches are reflected in the women’s narratives.

**Methods:**

Semi-structured interviews were conducted with 17 women who had participated in a lifestyle intervention for women with BMI ≥ 30 kg/m^2^ during pregnancy 3 years earlier. The interviews were digitally recorded and transcribed in full. Thematic analysis was used.

**Results:**

The meaning of changing lifestyle for minimising weight gain and of the professional’s care approaches is described in four themes: the child as the main motivation for making healthy changes; a need to be seen and supported on own terms to establish healthy routines; being able to manage healthy activities and own weight; and need for additional support to maintain a healthy lifestyle.

**Conclusions:**

To support women with BMI ≥ 30 kg/m^2^ to make healthy lifestyle changes and limit weight gain during pregnancy antenatal health care providers should 1) address women’s weight in a non-judgmental way using BMI, and provide accurate and appropriate information about the benefits of limited gestational weight gain; 2) support the woman on her own terms in a collaborative relationship with the midwife; 3) work in partnership to give the woman the tools to self-manage healthy activities and 4) give continued personal support and monitoring to maintain healthy eating and regular physical activity habits after childbirth involving also the partner and family.

## Background

Maternal obesity, defined as body mass index (BMI) ≥ 30 kg/m^2^, during pregnancy is increasing and related to life-threatening and ill-health conditions in both mother and child. It involves risks of morbidity for the mother and for the fetus to develop birth defects, premature birth, perinatal asphyxia and stillbirth [1]. Furthermore, pregnancy involves a risk for excess weight gain especially for women already overweight or obese [2]. If limited weight gain during pregnancy can be achieved, health outcomes for the mother can improve with lower risk of obstetric complications without elevated risks for the fetus [3]. Initiating and maintaining a healthy lifestyle during pregnancy (i.e. keep active and eat healthily) will improve health and decrease risks of long-term illness in mother, child and whole family [4–8]. The goal during pregnancy, according to the American Institute of Medicine (IOM) recommendations, is for the woman to achieve weight control [9–11]. For this purpose, effective and well-designed interventions directed to pregnant women with BMI ≥ 30 kg/m^2^ concerning lifestyle change are still needed [12–14].

Pregnant women with BMI ≥ 30 kg/m^2^ have described negative experiences of the professional care they have received during pregnancy and childbirth [15–17]. A negative experience of encounters with health care providers, including stigmatisation and a patronising approach can be a barrier for engaging with lifestyle support [17]. Behavioural change can be difficult to initiate, as deeply rooted lifestyle patterns can be highly resistant to change [18, 19]. However, pregnancy is in itself a crucial period for potential change to a healthier lifestyle due to maternal concerns for the expected child [20]. The support and the motivation that will work for each woman with obesity is unique so to find out what is suitable for her it is necessary to start from her personal circumstances and needs [21, 22].

To date there have been few published accounts of how pregnant women with high BMI experience weight management care pathways and interventions. These have mostly been conducted in the UK [23–25] but also in Sweden [22]. Collectively this research suggests that women who have chosen to take part in a weight management service view this as a positive experience [22, 24, 25] and that it has helped them with physical activity and healthy eating behaviour change [22, 24]. In Gothenburg, Sweden, the project ‘Mighty Mums’ [26] offering support for pregnant women with BMI ≥ 30 kg/m^2^ to adopt a healthy lifestyle was initiated in 2011, including both additional visits to the midwife as well as opportunity to meet a dietician, something previous interventions have lacked [26]. Compared to matched controls the women in the Mighty Mums intervention group had a significantly lower gestational weight gain and lower weight retention postpartum [26]. Furthermore, the results from the project show that care could be provided with only a small additional cost and guidelines built on the project have now been implemented as standard care in the region for women with a BMI ≥ 25 kg/m^2^ at antenatal health care enrolment. It has not yet been explored whether the programme affects the women in the long term and what the women experience as helping or hindering factors in the pregnancy care to maintain a healthier lifestyle. Therefore, the aim of this study was to explore the experiences of women with BMI ≥ 30 kg/m^2^ regarding minimising their gestational weight gain, and to assess how health professionals’ care approaches are reflected in the women’s narratives.

## Methods

### Participants and setting

This study was carried out in Gothenburg, Sweden. All interviewees had participated in an antenatal health care lifestyle intervention called ‘Mighty Mums’ aiming at a healthy amount of weight gain (below 7 kg) in pregnant women with BMI ≥ 30 kg/m^2^ 30 in the beginning of pregnancy three years earlier. The women were informed about the benefits, for themselves and the baby, of a gestational weight gain below 7 kg, and that the intervention could be helpful to reach this goal. The intervention comprised of regular weighing, two extra scheduled visits with the antenatal health care midwife, 5 min of advice and support of regular physical activity and healthy nutrition on regular follow-up visits, including exercise on prescription, pedometers, walking poles, aqua aerobics, individually adapted food plans and group or individual sessions with a dietician - all voluntary and free of charge [26]. The women were also informed about ongoing activities offered by community health centres and were actively encouraged to participate in these by the midwife.

Before the start of the project, all midwives in antenatal health care were offered education about obesity in pregnancy in general, and more specifically about nutrition and physical activity in pregnancies with maternal BMI ≥ 30. They also obtained training in motivational interviewing. During the project, the midwife could find information on a website for self-education, as well as pedagogical tools to work with and leaflets to give to the participating women. The women’s regular midwives were engaged in the project, and the project leader - a dietician - was available if there were questions on the procedure or on how to work with a logbook (a documentation of how and on what level the woman chose to participate and how the project proceeded on an individual basis). The ‘Mighty Mums’ project is described in more detail elsewhere [26].

Interviewees were contacted by telephone in connection with or after a follow-up meeting 2½-3 years after they participated in the ‘Mighty Mums’ project. They received oral and written information and were asked to participate in an interview and all gave written consent. Of a total of 45 eligible women who had participated in the study follow-up at 2½ years after childbirth, 19 women were approached and 17 agreed to participate. Purposive sampling was used to allow a mix of primi- and multiparous women of different age and country of birth. Characteristics of interviewees are shown in Table 1.

**Table 1:** Characteristics of study participants, *n* = 17

Age, median (range), years	35 (28–45)
Nulliparous/multiparous during project	12/5
Age of child in project at time for interview, years	2½-3½
Number of children, range	1-6
Had another child after project	4 women
Pregnant at time for interview	2 women
Born outside Europe	2 women
Gastric by-pass operation before project	3 women
BMI in early pregnancy, median (range), kg/m^2^	33.6 (30.1–41.1)
BMI at postpartum check-up, median (range), kg/m^2^, 2 missing	33.3 (28.8–40.9)
Self-reported BMI 2½ years postpartum, median (range), kg/m^2^, 1 missing	31.7 (27.5–38.5)

### Data collection

The interviewees were encouraged to give detailed narratives of their experiences, and a semi-structured interview guide was used to cover all fields of interest. The women were asked about their experience of participating in the lifestyle intervention during pregnancy 3 years earlier [26], whether they received the help and support concerning healthy eating and physical activity they needed during pregnancy, how the planning to achieve a healthy lifestyle was made, whether they had maintained the healthy habits after childbirth and to reflect on what further assistance they would have needed to maintain healthy habits in the long term. The interviews were performed by SD (*n* = 16) and AD (*n* = 1) from July 2014 to April 2015. The interviews took place in a location the interviewees chose (*n* = 7) or were conducted as telephone interviews (*n* = 10). They all lasted between 25 and 75 min, were conducted in Swedish and digitally recorded.

### Data analysis

All interviews were transcribed verbatim and analysed using thematic analysis [27]. The analysis was data-driven and performed inductively rather than driven by prior theoretical assumptions and was made separately by three researchers (AD, MB and ÅP) on the basis of the Swedish transcriptions. One researcher (ÅP) was familiar with the ‘Mighty Mums’ project and the two others (AD and MB) are midwives researching obesity in relation to childbearing. First, the text from all interviews was read in full to become familiar with the data. The first author (AD) used NVivo Version 10 to identify, condense and initially code all text features with a meaning. MB and ÅP did these steps manually. Next, using NVivo, codes were sorted into candidate themes in relation to the research question. The candidate themes were refined and adjusted to cover all meaning patterns in the text and to be coherent, enough distinct from other themes, and internally consistent. In the next step, these three researchers (AD, MB and ÅP) reviewed the potential themes in relation to the whole data set and adjustments were made. Last, final themes were defined and named to correspond to the essential meaning in each theme. The first author (AD) wrote the analysis text and chose the quotes. In a final phase the whole findings section was reviewed by the whole research group including midwives, a dietician, a gynaecologist/obstetrician, a health psychology researcher and a bachelor in global studies. The main data collector (SD) was not familiar with the ‘Mighty Mums’ project. In the final stage the findings text including quotes, was translated into English by two authors (SD and EKO).

### Findings

The meaning of changing lifestyle for minimising weight gain and of the professional’s care approaches is described in four themes: the child is the main motivation for making healthy changes; need to be seen and supported on own terms to establish healthy routines; being able to manage healthy activities and own weight; need for additional support to maintain a healthy lifestyle. Quotes are displayed in italics from the interviewees, called IP1-17.

### The child is the main motivation for making healthy changes

The expected child was described as the main motivation for a healthy lifestyle, both in terms of healthy eating and physical activity. The women saw their weight as their responsibility, if not caused by a disease, e.g. hypothyreosis. The women’s own health, which may have been neglected before, became more significant during pregnancy, due to the direct effect it had on the unborn baby. The women felt that the concern to provide the child with the best conditions possible helped to refrain from unhealthy food.

*When I got a craving for sweets or chocolate I said to myself “no, I won’t eat it” and that was for the sake of the baby growing inside me. It wasn’t for me. I tried to lose weight before but it didn’t work. So, now it wasn’t for me but for the baby inside me, and that was a much greater motivation.* IP5

Living with a high BMI was described by some women as a trauma and taboo including feelings of being judged by an unsympathetic environment. A stressful lifestyle and a demanding work environment stood as obstacles to prioritise own health. Low self-confidence and reluctance to show their body was described as barriers for exercising, and past failures to reduce weight hindered further attempts. Sometimes there was a lack of confidence in their own ability and circumstances to make lifestyle changes.

*It (being overweight) is still considered something shameful and most people think that you just have to stop eating and everything will be all right.* IP1

Despite awareness of the unhealthy habits, and what a healthy diet includes, it had been difficult to change to healthy habits before the pregnancy. A great awareness was present that the high BMI involved risks and the women were willing to accept help in order to minimise the gestational weight gain and make healthy lifestyle changes.

*Something got me motivated to change my diet very-very-very much. And it made me a mother who could cope.* IP17

During pregnancy the excess weight caused concern for the women related to the negative impact it might have on the parturition and the expected child. Even though the information concerning increased risks for the baby related to high BMI usually was a motivation for lifestyle change, it could also be perceived as frightening and make the woman feel that she risked the health of her unborn child.

*Yeah, I was a bit shocked by it all, that I carried so much extra weight, when we did the weighing. I didn’t really know… it was a bit overwhelming. That it was a danger to me and a danger to my child… “Huh!” stressful… I never needed much healthcare before and… now I was a concern, a possible danger to myself and my child. I felt like I was being labelled.* IP2

Several narratives of the women revealed a long history of overweight, sometimes since childhood. Others had gained major weight during a previous pregnancy. Some women believed that their genetic predisposition prevented weight loss, and had had gastric surgery or planned to have it. Changing for the baby was a strong motivation, superseding the previously perceived barriers and demotivating factors.

*I know that I have a predisposition to carry extra weight, and I do not want my children to end up in the same situation. If I can influence them to make healthy choices, so that they live well and healthily, I will of course do that.* IP8

### Need to be seen and supported on own terms to establish healthy routines

To build a trusting relationship with the midwife, the women argued that the midwife must show that she is interested, ask questions in an active way and listen to the woman. Such midwifery involvement made the woman feel secure and confident talking about what was important to her, where weight was one of many topics. The women often met the same midwife at each visit which provided continuity of care and facilitated the communication.

*You could say that you gain weight in a controlled way (in the Mighty Mums project). So that you don’t gain too much really fast. To share thoughts and ideas; what should I eat? How should I think? Am I eating wrong now?* IP8

Frequent visits where the midwife suggested and the women chose activities helped the women modify their weight management through healthy eating and physical activity during pregnancy. The women wanted to demonstrate good results on the planned lifestyle changes in the individual meetings with the midwife and get encouragement and positive feedback in return.

*To begin with I was supposed to get a routine for eating breakfast. And get routine eating properly in the first place. And to have set hours… managing it bit by bit. Starting by getting a routine for this and then for that. And that’s really good, rather than doing everything at once.* IP11

The commitment from the midwife could be limited by stress and lack of time and the visits sometimes felt time pressured for the woman without enough time for consultation, due to limited time for the visit. Some women felt pressure to contribute to get good results at the clinic and perceived the set weight goal (below 7 kg of maternal weight gain) as too difficult to achieve. Focus was then not primarily on the woman’s health, but to achieve good results within set healthcare targets.

*I think that my midwife was quite stressed and wanted good results to show; therefore she got a bit stressed when I continued to gain weight, although a lot of it was water… I felt pressure, a lot of pressure from outside… it wasn’t a lot of: “how are you?”* IP2

There were limitations to what kind of support that was possible to get, and some women said that they did not ask for support that they thought was not available. Some women followed the set plan and still gained weight, but there was not always interest to understand why and the women had to seek help elsewhere. The women who were not sufficiently listened to lost confidence in the health care providers and felt that they were badly treated.

*They didn’t take any tests, instead they assumed that… just assumed that I exercised too little and ate too much, but that wasn’t the case.* IP10

There was a need for the women to discuss weight with their healthcare provider in a non-judgmental manner. Training for healthcare professionals regarding how to initiate weight discussions was requested. Some women had had bad experiences of not being treated with respect by healthcare staff, with offensive comments about their appearance.

*She’s beating around the bush for like ten minutes and then it comes: “yeah, well you have to start thinking about what you eat, you’re pretty big” instead of, perhaps, talk about BMI and treat it like something normal. It is kind of a delicate issue… and pregnant people are not exactly known for being the most non-sensitive humans in the world (laughing). You are kind of sensitive.* IP1

The discussions during pregnancy focused on the women’s weight but not what caused this weight, which to some women was very important to discuss. Some women said that the healthy routines were likely to be temporary if you failed addressing the underlying problems and argued that there was a lack of both knowledge and commitment for discussing weight issues within the current healthcare system.

*Maybe you need to get to the core of the problem, why the situation looks like it does, and then you can start making changes… because nothing says, when the pregnancy is over, that you will continue if your mind-set hasn’t changed… for me it was about so much more than just getting started with exercise. It was about how I looked at myself and the way I valued myself and how I punished myself – or not punished myself – through food… It was about something much deeper than just working out. This is how I feel about it.* IP3

### Being able to manage healthy activities and own weight

Different tools and strategies introduced by the midwife helped the women control their weight during pregnancy. The tools referred to by the interviewees consisted of walking poles, pedometers, aqua aerobics, exercise on prescription, food diary, dietary advice, extra visits to the midwife and individual or group sessions with a dietician. At the antenatal visits they were able to discuss their lifestyle and reflect together with the midwife. Measuring own weight with a weighing scale was also used as a tool to gain control. To get a sense of control of the weight management led to more awareness, pride and joy for the women.

*When we had the follow-up in the project I felt that it was fun to do the weighing. If it went well it was fun but if you didn’t have a good result then it wasn’t fun either. But the thing is that you all the time, like… because the main thing is still not to gain too much weight and we saw that on the weighing scale when we did the weighing.* IP8

The group meetings with the dietician provided useful tips on regular meals and healthy snacks during pregnancy. There was also need for individual nutrition counselling on how an individual diet could be designed, and when experiencing difficulties relating to how to eat during pregnancy. To meet and discuss with others in the same situation gave a feeling of community.

*It was important to discuss with the other mums… the fact that you are not alone but there are others who experience the same thing.* IP12

Everyday exercise (for example walking or climbing stairs) in combination with - or instead of - group activities, was used and the women could discover small activities that fit into their own life leading on to more physical activity. The aqua aerobics was considered a fun and gentle exercise where the body felt almost weightless and a good activity when suffering pelvic girdle pain or body aches and which also contained exercises that were preparatory for childbirth.

*I have never met a leader who was as committed as her (the aqua aerobics leader). And that was quite wonderful to see because there were a few mums that where quite heavily pregnant, if you say so. The largest bellies I’ve ever seen that bounced around in the water. It was a lovely… you got a huge energy boost out of it, which lasted longer than for just that moment when you where there.* IP9

Some women experienced that there was an uncertainty in planning and goalsetting and had wished for a more strict approach to weight management. Some women chose instead to manage the planning of their lifestyle change at home with the support of their partner.

*Especially when it came to physical activity he (partner) was very important. Because I’d asked him to encourage me and to be positive and to set up and prepare my exercise machine so that when I got home I couldn’t dodge it (laughing).* IP6

### Need for additional support to maintain a healthy lifestyle

The extra support during pregnancy ended when the women needed it the most. The follow-up after childbirth was limited or absent and the healthcare focus shifted to the child. The women experienced that they were not prepared to face new situations with the child and how to handle food after they stopped breastfeeding. Coping with own work situation, stress, keeping busy with child care and the relationship with the partner were other barriers for keeping healthy habits in the long term. The strong motivation gained during pregnancy decreased when the woman no longer was directly connected to her child.

*Suddenly, it was about taking care of myself for my own sake … so then it was like sweets and less healthy food, not as much salad, I mean, the bad things came back in a way.* IP7

Some women were disappointed with their own effort and thought that they could have asked for more specific help during pregnancy, and now they find themselves back in the same situation as before the lifestyle intervention. As the women sometimes assumed that the health care providers would know what was needed, they accepted the offer given without further reflection, suppressing own opinions and personal beliefs. Sometimes the project was perceived as a fixed concept not adaptable to individual needs.

*In the beginning you could get some talks and the opportunity to adjust to the situation. I mean get the person on track instead of run into action right away, so to speak. To get the chance to come to terms with the whole thing and also to be a bit more flexible: “what may work for you?”* IP2

Nonetheless, for some women the healthy eating habits during pregnancy and the everyday exercise stood as a beginning of a maintained lifestyle change. The women tried to find other alternatives to food that could serve as reward and strategies to make time for physical activity, putting the knowledge of healthy habits into practice.

*What was best for me anyway was that you recognise and become aware of the importance of getting started with the little things, so to speak, to keep the body in motion. It doesn’t have to be workout in the sense of going to the gym or so; but daily physical movement, that you implement this more and more.* IP13

Health was described as an active choice where it was more important to achieve a weight that was healthy than being skinny. The women had joined the lifestyle project without hesitation because they felt that they did not have anything to lose and because they were prepared to change their lifestyle. Some of the women had already begun losing weight before pregnancy and for those women the intervention worked as support to continue on this path.

*To stay healthy is work. You have to do exercise and… I mean it’s not that motivating to go to the gym every day… it takes an effort. Staying at home watching soaps and play with your bellybutton, that’s more comfortable, it’s not that big of an effort, but it is more harmful. It is a choice you do, an active choice.* IP7

To maintain a healthy lifestyle became more difficult in the long run and the women missed having someone who was there to do follow-ups, to motivate and provide feedback in lifestyle issues, on how to maintain good eating habits and strategies for exercise. There were insights regarding the necessity of finding balance in order to maintain a healthy weight and that has to be a long-term goal because it takes time to change thoughts and behaviour.

*I’m thinking like this that maybe you could have, through your medical centre, an appointment once a month. Because then you would know that you were going to be checked on and then perhaps you would have had a bit better focus on how to take care of yourself … I function like that, when I have some work assignment for which I have the responsibility, then that requires all my brain capacity and then it won’t leave much time or planning for taking care of yourself.* IP6

## Discussion

The results show in four themes how women with BMI ≥ 30 kg/m^2^ experience participation in a lifestyle intervention during pregnancy and how they reflect on how this participation affected them three years later. The main motivation for attendance in the lifestyle intervention was to provide good conditions for the child, both in the womb, and with healthy habits in the family. The women had been living with a high weight before pregnancy and sometimes since childhood. Although they had knowledge of how to eat healthily the expected child became the main motivation for changing lifestyle. The women needed to get personal support from the health care providers and receive help to be able to control the selection and implementation of healthy activities. Extra support during pregnancy helped temporarily but there was still a need of support to maintain a healthy lifestyle in the long run, suggesting pregnancy is not necessarily a ‘window of opportunity’ for long-term, but rather for short-term behaviour change. Pregnancy as a window of opportunity is naturally used for motivation [28], but can provocatively be seen as an automatic mantra of the health care professionals without long-term meaning. Some, not all, women clearly expressed that they felt abandoned postpartum, although for others this window of opportunity seems to have worked for the long run.

In contrast to previous research [19, 25] the interviewees in this study had received information and were aware about the risks with the high BMI for themselves and their unborn babies. Due to this knowledge, the women could feel anxious and have feelings of being a danger to the child even though the information and awareness of the risks was a motivating factor. The women wanted clear and factual information and help to adopt a healthier lifestyle for the sake of the baby, corresponding to earlier research where motivation for lifestyle change during pregnancy are described [23, 29]. Women with BMI ≥ 30 kg/m^2^ often report a long struggle with their weight and difficulties to lose extra weight after an earlier pregnancy [30], which also applies to the women in this study. Living with a high BMI was experienced negatively, similar to descriptions in other studies [31]. Contrasting with some earlier research [29] our interviewees considered pregnancy as an opportunity to avoid excessive weight gain. The reason for this fact could be due to the design of the intervention project where the women were informed of health benefits of limited gestational weight gain for own health, lower risk facing the birth and better outcomes for the baby [26]. This approach is more motivating in a positive sense rather than an intervention where people are simply given risk information to frighten them into change.

The theme of managing weight for the sake of the baby suggests, however, that even if the women are given the information about health benefits to themselves they may not experience this as motivating in the same manner. This is in line with a range of sociological literature that has discussed the moral burden on mothers, in which the need to fulfil the role of a good mother is stronger than the perceived need to protect one’s own health. Social and cultural attitudes towards motherhood, in addition to gender constructs, mean that women perceive a strong need to present themselves as good mothers and experience considerable stigma if they are socially perceived as not fulfilling such a role – such as by having a normal weight or by adopting normative choices in pregnancy, around birth and in their mothering [32].

Central in the women’s stories was the need to get personal attention and support on their own terms. They wished that the issue of weight was discussed in a straightforward and non-judgmental way by health professionals because it is a sensitive issue [16, 24, 33, 34]. The women preferred professionals to refer to BMI when discussing weight [23] and the interviewees also requested training of health care providers in how to approach the subject and counsel them, which is consistent with previous qualitative studies showing that health care staff face difficulties in communication about weight and weight management during pregnancy with women with high BMI [14]. Furthermore, our results underpin earlier findings of the stigmatisation of obesity where attitudes among health care providers, guidelines, written information and how weight discussions are initiated and performed need scrutinising not to include hidden stigma messages [35].

Some of the interviewed women reported that the health care providers lacked interest in their personal well-being. Instead they could feel being reduced to a weight issue with the purpose of getting good results at the antenatal care clinic. This approach is opposite to the one described by Ekman et al. as person-centred care (PCC) [36] where PCC reduces the risk of depersonalisation. Instead, the core elements in person-centred care include listening to the person’s narrative, working in partnership and safeguarding the partnership through documentation [36]. Our results confirm earlier studies showing that women with a high BMI may feel badly treated in health care [31] and support previous arguments for PCC in antenatal care of women with BMI ≥ 30 kg/m^2^ [21, 37]. The current study finding that the women wanted to be listened to and be met with respect in a personal relationship with the midwife corresponds to initiating the partnership in person-centred care [36]. Furthermore, focusing solely on the importance of weight in itself could pose a risk for people who have obesity to neglect health exams or screenings due to the stigmatisation [17]. For health care it is therefore important to emphasise increased health, instead of merely weight management, to provide good help and support to suit the individual woman [25, 31]. In addition, this may help to improve the focus on the women’s positive health gains, given that the theme of motivation for the sake of the unborn child may not encourage *long-term* weight management and health benefits for the women.

Help and support to be able to choose healthy activities was described as important. An approach that suited the women well was when in discussions with the midwife, she suggested options, and the woman decided which to aim for. This compares well with the routine of working the partnership in PCC [36]. This partnership is described in these interviews as discussing together what is desirable and at the same time realistic and in collaboration making a plan that takes into account the woman’s own situation. Working in partnership then means that the women receive knowledge and help to manage information to be able to make informed decisions and find activities that promote health and that suit their own situation. This gives the woman the tools to manage her situation of her own accord and being less subjected to conflicting advice and general prescriptions about pregnancy [14, 18, 21, 37].

Our results indicate that follow-up appointments are needed to maintain healthy routines over time. This is consistent with previous research that has shown that the effect decreases when the intervention ends [23, 25]. Continued health care support for the woman after pregnancy can give positive health effects for her and her family. Lifestyle interventions during pregnancy is most often targeting the pregnant woman only and not her partner or family who have a great influence over her habits [14, 29]. To be truly meaningful the help and support to the woman should give her the tools to self-manage her situation and also involve her partner and family. In the lifestyle intervention described here the women completed food diaries and the midwife made notes about the lifestyle activities in a logbook [26]. Our interviewees did not talk specifically about documentation but about the importance of follow-up meetings. Therefore, safeguarding the partnership according to PCC [36] can be understood as providing continued support to maintain a healthy lifestyle after childbirth in the care of women with BMI ≥ 30 kg/m^2^.

The woman and her family are living in a context that cannot be separated from the individual, with opportunities to consume food and beverages at convenience and inexpensively. In the modern society it is easy to obtain a lot of energy with little of nutritional value [38] and therefore approaches that simply target individual behaviour without recognising the social context may not be as effective as they optimally could have been. There are also barriers to physical activity, and some of them reported by women are scarcity of time and lack of energy [38]. Furthermore, a longer distance to open green space areas, which is often the case in a city, will render it more difficult taking walks [39]. The driving force for the increasing prevalence of overweight and obesity is the obesogenic environment, and it is important to have this in mind when dealing with individuals with difficulties to keep their weight within healthy limits [40]. It would be of advantage for the woman and her family if the society was more oriented towards health, and more helpful when it comes to making healthy everyday choices, e.g. marketing of healthy/unhealthy food and supporting healthy alternatives, decreasing servings and package sizes, facilitating safe walking in green areas close to home, promoting architecture that makes it easier to take the stairs, and similar measures. A clear stance for health on the whole life span is of uttermost importance, and women need to universally be reached by the same health message – in health care, school, work, media and public places.

### Strengths and limitations

Women who declined to participate in the lifestyle intervention are not accounted for, which is a limitation since interviewing pregnant women about why they chose to decline weight management support can help improve said intervention [41, 42]. Another limitation of this study is that the small sample size (17 women) may limit the applicability of these results. Two approached women declined to participate and may have had other views. Professional background of researchers may influence data interpretation. This risk of bias was reduced by a mixture of professions in the group, with experience both within and outside the health care system. The interviewees were encouraged to speak freely in a place of their own choice and the interviewers were not involved in delivering the intervention. This together with the length of the interviews was considered to reduce the risk of social desirability. Almost all of the contacted women agreed to be interviewed (17/19). Likewise, the first author had no experience of the intervention and therefore less risk of influencing the results. Strengths also include that the interviews were performed three years after the intervention and the women could reflect upon how their day-to-day behaviour was affected after these years. The purposive sampling of participants allowed a mix of primi- and multiparous women of different age and countries of birth and therefore the results may be representative for Swedish women.

## Conclusions

Our findings have implications for how services are organised to support women with BMI ≥ 30 kg/m^2^ to gain a healthy lifestyle during pregnancy and postpartum. First, the health care providers should address women’s weight in a non-judgmental way using BMI, not make assumptions about their diet and activity levels or their motivations and difficulties and give accurate information about the benefits of a limited gestational weight gain and the risks of obesity in accurate and manageable manner. Second, that the women want support on their own terms in a personal relationship with the health care provider (midwife). Third, working in a partnership regarding lifestyle changes during pregnancy gives the woman the tools to manage choosing and performing healthy activities. Support is also needed postpartum to aid long-term behaviour change. Furthermore, these results contribute to the understanding of what is important for women’s lifestyle in the long term, i.e. that the strong motivation to change for the sake of the child may subside after childbirth, and the need of continued personal support and monitoring to maintain healthy eating and exercise habits. Therefore, the help and support of the woman should also involve her partner and family and optimally include referral to community health centres or lifestyle receptions at primary care level.

## Abbreviations

BMI, body mass index
